# Detection of Extended Spectrum ß-Lactamase-Producing *Escherichia coli* with Biofilm Formation from Chicken Meat in Istanbul

**DOI:** 10.3390/foods13071122

**Published:** 2024-04-07

**Authors:** Ali Aydin, Ali Anil Suleymanoglu, Abzal Abdramanov, Peter Paulsen, Emek Dumen

**Affiliations:** 1 Department of Food Hygiene and Technology, Faculty of Veterinary Medicine, İstanbul University-Cerrahpaşa, 34320 Istanbul, Türkiye; alianil.suleymanoglu@ogr.iuc.edu.tr (A.A.S.); emekdumen@iuc.edu.tr (E.D.); 2 Department of Veterinary Sanitary Examination and Hygiene, Kazakh National Agrarian Research University, 050010 Almaty, Kazakhstan; abzal.abdramanov@kaznaru.edu.kz; 3 Unit for Food Hygiene and Technology, Centre for Food Science and Veterinary Public Health, Clinical Department for Farm Animals and Food System Science, University of Veterinary Medicine Vienna, 1210 Vienna, Austria

**Keywords:** antibiotic susceptibility, biofilm, carbapenem resistance, *Escherichia coli*, ESBL, *mcr*

## Abstract

Antimicrobial resistance is one of the major public health problems worldwide. This study aimed to detect the presence of extended-spectrum β-lactamase-(ESBL-)producing *Escherichia* (*E.*) *coli* in chicken meat in Istanbul, Türkiye. Raw chicken meat samples (*n* = 208) were collected from different sale points and analyzed for ESBL-producing *E. coli*. In total, 101 (48.5%) isolates were confirmed as *E. coli* by PCR, of which 80/101 (79.2%) demonstrated multiple antibiotic resistance. Resistance against amoxicillin-clavulanic acid was most frequent (87.1%). Eighteen isolates (17.8%) demonstrated phenotypical ESBL resistance, as assessed by the double disc synergy test (DDST). Isolates were tested for the presence of β-lactamase genes and mobilized colistin-resistant genes. The *bla*_TEM_ group was most frequently detected (97.02%), followed by *bla*_CTX m_ (45.5%), *bla*_SHV_ (9.9%), and *bla*_OXA-2_ (0.9%). However, *mcr* genes and *bla*_NDM,_ *bla*_KPC_, *bla*_VIM_, and *bla*_OXA-48_ genes were not found in any isolate. *E. coli* strains were tested for biofilm formation in six different media [Nutrient broth, LB broth, Tryptone Soya broth (TSB), TSB containing 1% sucrose, TSB containing 0.6% yeast extract, and BHI]. Biofilm formation by *E. coli* isolates (44/101, 43.5%) was highest in TSB with 1% sucrose. It is worth noting that all biofilm-producing isolates were found to harbor the *bla*_TEM-1_ gene, which can indicate a high level of antibiotic resistance. This is the first report about ESBL-producing *E. coli* in poultry meat, the exposure of consumers in Istanbul metropolitan areas, and the ability of *E. coli* from this region to produce biofilms.

## 1. Introduction

Antimicrobial resistance (AMR) is a major public health concern worldwide, leading to the clinical failure of antimicrobial therapy. Poultry production worldwide uses substantial amounts of antibiotics, and there are concerns about high AMR levels among bacteria isolated from poultry samples. The frequency of AMR and genes causative for AMR in *Escherichia coli* (*E. coli*) isolates are reported from many different countries [1,2], including Türkiye [3,4]. A one-health approach is required to control the emergence and effects of antibiotic resistance [5]. In this context, multiple drug-resistant (MDR) *E. coli* in chicken meat are of concern, with implications for human consumers, the health of animals, and the environment [6]. The rapid emergence of multidrug-resistant *E. coli* strains has resulted in human morbidity and even fatalities [7].

Beta-lactam antibiotics represent one of the major classes of antimicrobials. Emerging antibiotic resistance has compromised their antibacterial efficacy [8]. In some bacteria, particularly Gram-negative bacteria, beta-lactamases have evolved, i.e., enzymes that can break down beta-lactam antibiotics. The expression of beta-lactamases is one of the most studied and widespread mechanisms of antimicrobial resistance [8]. ESBL producers have been identified mainly in the taxonomic order *Enterobacterales*. These bacteria can harbor multiple determinants of antibiotic resistance, making it more difficult to treat infections caused by these pathogens [9]. *Enterobacterales* producing ESBL are thought to have colonized more than 1.5 billion individuals around the world, essentially in devastated countries and also in industrialized countries. Furthermore, ESBL producers have complex epidemiology, most prominently *E. coli* and *Klebsiella pneumoniae*, whose reservoirs include the environment (soil and water), wildlife, livestock, food, and pets [10].

*E. coli* that produces ESBLs has been identified as a major multi-resistant pathogen associated with serious hospital- and community-acquired infections worldwide, particularly where sanitation and hygiene practices are poor or lacking [11]. The European Food Safety Authority (EFSA) has identified ESBL/AmpC-producing *E. coli* as one of the main priority hazards, especially in poultry. In various studies, ESBL-producing *E. coli* have been isolated in broiler farms and slaughterhouses, concluding that chicken meat is a potential source of infection for humans [12]. The source and transmission methods of ESBL-producing *E. coli* strains have yet to be fully explored. However, over the last decade, more research on ESBL-producing *E. coli* in animals and animal feeds has been undertaken in Europe [13,14].

Biofilms generally comprise extracellular polymeric substances that enable bacteria to adhere to surfaces and communicate with each other [15]. Biofilms are described as the most widespread and most successful life forms on Earth. Quorum sensing (QS) alters the gene expression of bacteria according to the size of the bacterial community formed by the biofilm. The production of antimicrobial proteins can be promoted by QS, which can lead to increased antimicrobial resistance. Moreover, the matrix formed by biofilms facilitates the transfer of antibiotic resistance genes [16]. It has been suggested that beta-lactamases can be secreted from bacteria into the surrounding biofilm matrix and, thus, into the environment [17].

The aims of this study were: (a) to examine the presence of *E. coli* in chicken samples collected in the Asian and European part of metropolitan Istanbul with conventional and molecular genetic methods (PCR); (b) to determine phenotypic ESBL-producing *E. coli* strains; (c) to detect biofilm-producing *E. coli* in six different media (Nutrient broth, LB broth, Tryptone Soya broth, TSB containing 1% sucrose, TSB containing 0.6% yeast extract, and BHI); and (d) to investigate ESBL (*bla*_SHV_, *bla*_TEM_, *bla*_CTX-M_, and *bla*_OXA_), carbapenem (*bla*_VIM_, *bla*_OXA-48_, *bla*_ND_M, and *bla*_KPC_) and mobilized colistin (*mcr*-1, *mcr*-2, *mcr*-3, *mcr*-4, *mcr*-5, *mcr*-6, *mcr*-7, and *mcr*-8) as resistance genes in these *E. coli* strains.

## 2. Materials and Methods

### 2.1. Sampling 

In total, 208 raw chicken meats were collected from different sale points (market, butcher) from May to August 2021 in Istanbul, Türkiye. Half of the samples were collected from the European side of Istanbul [drumsticks (*n* = 14), breasts (*n* = 25), thighs (*n* = 25), and wings (*n* = 40)]. The other 104 samples were sampled from the Asian side [drumsticks (*n* = 14), breasts (*n* = 34), thighs (*n* = 27), and wings (*n* = 29)]. All samples were transported in thermal boxes at ≤+4 °C to the laboratory (Department of Food Hygiene and Technology, İstanbul University-Cerrahpaşa) and were processed immediately upon arrival.

### 2.2. Isolation and Identification of E. coli by Conventional Methods

The isolation and identification of *E. coli* were conducted according to the ISO 16649-2 standard method [18]. Several pieces were taken from each sample to give a weight of 10 g. To this 10 g, 90 mL of Buffered Peptone Water (Oxoid CM 0509, Basingstoke, UK) was added, and the suspension was mixed in a stomacher (Interscience, Saint Nom la Bretèche, France). Subsequently, Tryptone Bile X Glucuronide Agar (TBX; Oxoid CM 0945) was inoculated and incubated at 41 ± 1 °C for 24 h. Suspected (green) *E. coli* colonies on TBX agar were transferred onto Eosin Methylene Blue Agar (EMB; Oxoid 0069B) plates, which were incubated at 37 °C for 24 h for verification.

After the isolation, *E. coli* strains were plated for purity testing, and a single colony was streaked onto Tryptone Soya Agar (TSA; Oxoid CM 0131) from EMB and incubated at 37 °C for 24 h. Isolated strains were frozen in 20% glycerol stocks (Sigma G5516, Sigma Aldrich, Darmstadt, Germany) and stored at −20 °C for further analysis.

### 2.3. Verification of E. coli Isolates by PCR

#### 2.3.1. DNA Extraction

*E. coli* strains were cultured on Tryptone Soya broth (TSB; Oxoid CM 0129) at 37 °C for 24 h. In total, 750 µL from this enrichment culture broth was transferred into Eppendorf tubes (2 mL) and centrifuged at 10,000 rpm for 5 min. Then, the bacterial pellet was resuspended in TE (10 mM Tris-HCl pH 8.0 and 1 mM EDTA) containing lysozyme (Sigma 7651) and incubated at 37 °C for 18 h. In the next stage, 250 µL of 10% SDS and 20 µL of 20 mg/mL proteinase K (Sigma P2308-100MG)/dH_2_O were added into the Eppendorf tube, and the tubes were incubated at 56 °C for 2 h. Then, 750 µL of phenol/chloroform/isoamyl alcohol (25:24:1) (Amresco K169, Solon, OH, USA) was added, and the tubes were centrifuged at 14,000 rpm for 15 min. The supernatant was transferred into another Eppendorf tube. First, 150 µL of 5 M NaCl and then 700 µL of 2-propanol (Merck 1096342511, Darmstadt, Germany) were added for precipitating DNA, followed by centrifugation at 14,000 rpm for 10 min. Then, the supernatant was removed, and the pellet was washed twice with 1 mL of 80% cold ethanol (Sigma 459844) and centrifuged at 14,000 rpm for 10 min [19]. The pellet was resuspended in 75 µL of ultra-pure water. The acquired DNA was tested by BioTek Epoch2 (Agilent, Santa Clara, CA, USA) for its quality and stored at −20 °C. 

#### 2.3.2. Confirmation of *E. coli* Isolates by PCR (16S rRNA)

The identification of *E. coli* was performed using PCR. ECO-1 (5′-GACCTCGGTTTAGTTCACAGA-3′) and ECO-2 (5′-CACACGCTGACGCTGACCA-3′) (585 bp), which are specific primers to *E. coli*, were used in PCR [20]. The PCR assay was conducted with the following ECO-1 and ECO-2 conditions: initial denaturation at 95 °C for 5 min, 35 cycles of 95 °C for 30 s, 55 °C for 45 s, and 72 °C for 45 s. Subsequently, the PCR products were resolved on 1–1.5% (*w*/*v*) agarose gels in a 1 × TAE (Tris-acetate EDTA) buffer. The bands in the agarose gels were visualized using the SafeView™ Classic stain (ABM, Richmond, BC, Canada) in the Infinity Gel Imaging System (Vilber Lourmat, Marne-la-Vallée, France).

### 2.4. Antibiotic Susceptibility Tests in E. coli Strains

#### 2.4.1. Phenotypic Determination for Antibiotic Susceptibility in *E. coli* Strains

##### Screening for Antibiotic Susceptibility using Disc Diffusion Tests

All 101 confirmed *E. coli* strains were tested for antibiotic susceptibility by the agar disc diffusion method on a Mueller–Hinton Agar (MHA; Oxoid CM 337), according to the European Committee on Antimicrobial Susceptibility Testing (EUCAST) [21]. Disc diffusion agar test was performed on MHA for the following 16 different antibiotics: ampicilin (AMP; Oxoid, CTOOO3B, 10 µg), amoxicillin clavulanic acid (AMC; Oxoid, CT0223B, 30 µg), aztreonam (ATM; Oxoid CT0264B, 30 µg), cefotaxime (CTX; Oxoid, CT0166B, 30 µg), tetracycline (TE; Oxoid, CT0054B, 30 µg), ciprofloxacin (CIP; Oxoid, CT0425B, 5 µg), nitrofurantoin (F300; Oxoid, CT0036B, 300 µg), amikacin (AK; Oxoid, CT0107B, 30 µg), ceftazidime (CAZ; Oxoid, CTO412B, 30 µg), trimethoprim-sulfamethoxazole (SXT; Oxoid, CT0025B, 1,25 µg–23,5 µg), gentamicin (CN; Oxoid, CTOO24B, 10 µg), cefoxitin (FOX; Oxoid, CT0119B, 30 µg), chloramphenicol (C; Oxoid, CT0013B, 30 µg), cefuroxime (CXM; Oxoid, CT0127B, 30 µg), piperacillin/tazobactam (TZP; Oxoid, CT0725B, 36 µg–110 μg) and meropenem (MEM; Oxoid, CT0774B, 10 µg). Petri dishes were evaluated after 18 ± 2 h of incubation at 35 ± 2 °C, and *E. coli* strains were established to be sensitive or resistant following the EUCAST [22] and the Clinical and Laboratory Standards Institute (CLSI) [23] guidelines that defined the zone diameter breakpoints for each antimicrobial agent tested. For tetracyclines, breakpoints were provided only by CLSI [23] but not by EUCAST [22].

##### Detection of ESBLs Using Double Disc Synergy Test

For the phenotypic confirmation of ESBL in *Enterobacterales*, the double disc synergy test was used. For this test, paired discs of CAZ (30 µg) and CTX (30 µg) were used, and each was positioned at distances of 20 mm (center to center) from the AMC disc (AMC, 20 + 10 µg) [24].

#### 2.4.2. Genotypic Determination of Antibiotic Resistance Genes in *E. coli* Strains

##### Determination of ESBL Genes in *E. coli* Strains

A PCR assay was conducted to determine whether the isolates (101 *E. coli*) harbored *bla*_SHV_, *bla*_TEM_, *bla*_CTX-M_, and *bla*_OXA_. The PCR mix was as follows: 2.5 µL of DNA samples, a 10× KCL buffer at 2.5 µL, a dNTP mix (dATP, dCTP, dGTP, and dTTP) at 2.5 µL, MgCl_2_ at 1.5 µL, each primer at 0.5 µL, Taq DNA polymerase (Thermo Fisher EP0404; Thermo Fisher Scientific, Waltham, MA, USA) at 0.4 µL and dH_2_O at 12 µL, to give a final volume of 25 µL. A multiplex PCR to detect ESBL’s genes was applied, and initial denaturation at 95 °C for 15 min was followed by 30 cycles of 94 °C for 30 s, 62 °C for 90 s, and 72 °C for 60 s, with a final extension at 72 °C for 10 min in the thermal cycler (Veriti; Applied Biosystems, Waltham, MA, USA). The amplified PCR products were subjected to electrophoresis at a 1.5% agarose gel with the addition of 5 µL of safe view (ABM, Richmond, BC, Canada) (Table 1).

**Table 1:** Primers used for the detection of different β-lactamase genes by multiplex PCR.

Amplicon	Primer Sequence (5′→3′)	Band Size	Reference
*bla*_SHV_	5′-CTTTATCGGCCCTCACTCAA-3′ 5′-AGGTGCTCATCATGGGAAAG-3′	237	Fang et al. [25]
*bla*_TEM_	5′-CGCCGCATACACTATTCTCAGAATGA-3′ 5′-ACGCTCACCGGCTCCAGATTTAT-3′	445	Monstein et al. [26]
*bla*_CTX-M_	5′-ATGTGCAGYACCAGTAARGTKATGGC-3′ 5′-TGGGTRAARTARGTSACCAGAAYCAGCGG-3′	593	Boyd et al. [27]
*bla*_OXA_	5′-ACACAATACATATCAACTTCGC-3′ 5′-AGTGTGTTTAGAATGGTGATC-3′	813	Quellette et al. [28]

##### Detection of Carbapenem Resistance Genes in *E. coli* Strains

The PCR assay was conducted to determine whether the isolates contained *bla*_VIM_, *bla*_OXA-48_, *bla*_NDM_, and *bla*_KPC_ genes via a PCR assay using specific primers for each *E. coli* isolate (101 *E. coli*) according to the references (Table 2). The composition of the PCR mix was as follows: 3 µL of DNA samples, a 10× KCL buffer at 2.5 µL, a dNTP mix at 2.5 µL, MgCl_2_ at 1.5 µL, each primer at 0.5 µL, Taq DNA polymerase at 0.14 µL, and dH_2_O at 12 µL. The final volume was 25 µL.

Monoplex PCR was applied to detect carbapenem resistance genes, with the following conditions: initial denaturation at 94 °C for 5 min, followed by 30 cycles of 94 °C for 30 s, 30 s at the specific melting temperature given in Table 2, then 72 °C for 60 s, and a final extension at 72 °C for 10 min in the thermal cycler. The amplified PCR products were subjected to electrophoresis with 1.5% agarose gel and an addition of 5 µL of safe view (ABM, Richmond, BC, Canada) [29] (Table 2).

##### Detection of *mcr* Genes in *E. coli* Strains

The PCR assay was conducted to determine whether the isolates (101 *E. coli*) contained *mcr*-1, *mcr*-2, *mcr*-3, *mcr*-4, and *mcr*-5 genes. Conditions of multiplex PCR to detect *mcr*-1, *mcr*-2, *mcr*-3, *mcr*-4, and *mcr*-5 genes were as follows: initial denaturation at 95 °C for 15 min, followed by 30 cycles of 94 °C for 30 s, 58 °C for 90 s, and 72 °C for 60 s, and a final extension at 72 °C for 10 min in the thermal cycler (Table 3).

The PCR assay was conducted to determine whether the isolates harbored *mcr*-6, *mcr*-7, and *mcr*-8. The monoplex PCR conditions to detect *mcr*-6, *mcr*-7, and *mcr*-8 genes conditions were as follows: initial denaturation at 95 °C for 15 min, followed by 30 cycles of 94 °C for 30 s, 90 s at the specific melting temperature given in Table 4, and 72 °C for 60 s; the final extension was conducted at 72 °C for 10 min in the thermal cycler (Table 4). The amplified PCR products were subjected to electrophoresis using 1.5% agarose gel with an addition of 5 µL of safe view (ABM, Canada) for *mcr*-6 [30], *mcr*-7 [31] and *mcr*-8 genes [32].

### 2.5. Biofilm Formation Capability in E. coli Strains

The microplate method was used to examine the ability of *E. coli* to form biofilms with different media [33]. We studied the following six different media: Tryptone Soya broth (TSB), Tryptone Soya broth containing 1% (*w*/*v*) glucose, Yeast Extract, Brain Heart Infusion Broth (BHI; Oxoid CM 1135), Nutrient broth (NB; Oxoid CM0001), and LB broth (Miller) (LB; Merck 110285, Darmstadt, Germany).

*E. coli* isolates were incubated overnight (12–18 h) on TSB Agar at 37 °C. Then, the overnight cultures were adjusted to a density of 0.5 of McFarland and pipetted into microplates (3599 Corning Costar; Corning, NY, USA) with different media, followed by incubation at 37 °C for 24 h. After incubation, absorption was measured using a microplate reader (BioTek Epoch; Agilent, Santa Clara, USA) at 600 nm. The microplates were washed three times for biofilm detection as follows: 0.9% NaCl, methanol, crystal violet (Merck 1159400, Darmstadt, Germany), and 33% acetic acid (Merck 159166, Darmstadt, Germany) to remove the excess stain. After drying, the microplates were incubated with 5 mL of 96% ethanol for 15 min. The optical density (OD) at 595 nm was measured with a microplate reader (Bio Tek Epoch; Agilent, Santa Clara, USA) [34].

## 3. Results and Discussion

### 3.1. Detection of E. coli in Chicken Meat Samples in Istanbul

In total, 208 chicken meat samples were analyzed for *E. coli*, and 101 (48.6%) isolates were identified as *E. coli* by conventional microbiological methods and confirmed by PCR. In the chicken samples from the European side of Istanbul, 11 *E. coli* strains were detected in the drumstick (11/14; 78.5%), 14 strains in the breast part (14/25; 56%), 11 strains in the thigh (11/25; 44%), and 22 strains in the wing part (22/40; 55%). In the samples from the Asian side of Istanbul, 8 strains were detected in drumsticks (8/14; 57.1%), 14 strains in the breast (14/34; 41.1%), 11 strains in the thigh (11/27; 40%), and 22 strains (22/29; 37.9%) in the wing part. 

Chicken meat is widely processed and served in fast-food establishments and restaurants [35]. Therefore, poultry meat comprises about two-thirds of the total meat production in the world. Poultry meat production in Türkiye amounted to 2,245,770 tons in 2021, making Türkiye the world’s seventh largest chicken meat exporter in 2021 [36].

Baran et al. [3] obtained *E. coli* isolates from 105 (70%) of 150 chicken thighs sampled in Erzurum, Türkiye. In the present study, a lower percentage (48.5%) of *E. coli* was isolated when only chicken thighs were considered. In contrast, Guven and Kizil [4] reported a prevalence of *E. coli* of merely 7% (7/100) in chicken breast samples originating from Ankara, Türkiye. These results were lower than our findings (27.7%). The differences may be attributable, in part, to the continuous temperature control of refrigerators or ambient temperature in supermarkets [35].

### 3.2. Phenotypic Determination for Antibiotic Susceptibility in E. coli Strains

All 101 *E. coli* isolates were tested for susceptibility against 16 antibiotics of ten classes of antimicrobials. The antimicrobial susceptibility of the 101 *E. coli* isolates is shown in (Table 5). The highest resistance level was observed against AMC30, i.e., 87.1% (according to EUCAST [22]) and 67.3% (according to CLSI [23]). The phenotypic resistance of *E. coli* isolates was also frequently noted against AMP (79/101; 78.2%), TE (75/101; 74.2%), C (60/101; 59.4%), and SXT (53/101; 52.4%) (the results are based on CLSI [23] breakpoints). On the other hand, *E. coli* strains rarely demonstrated resistance to F300 at 1.9% and 0.9%, with 0% and 6.9% resistance to AK and 5.9% and 11.8% resistance against FOX, according to CLSI [23] and EUCAST [22], respectively. 

None of the isolates was resistant to TZP. Resistance to CTX, MEM, CIP, AMP, and SXT was higher in *E. coli* from chicken meat samples taken from the European side of Istanbul. In comparison, resistance to CN, FOX, CAZ, CXM, ATM, F300, and C was higher in *E. coli* from chicken meat samples taken from the Asian side of Istanbul. This difference in antibiotic resistance was the most pronounced regarding resistance against MEM (Table 6).

In a previous study [4], *E. coli* isolated from chicken meat in Ankara, Türkiye, were tested for phenotypic AMR by the disc diffusion test. All isolates were found to be resistant against TE. For AMP and CIP, AMR was found in 85.7% of the isolates; for C, SXT, CAZ, CTX, MEM, and CN, the corresponding figures were 71.4%, 42.8%, 28.5%, 28.5%, 28.5%, and 14.2%. A study conducted in Hatay, Türkiye, demonstrated similar results, with 91.4% of *E. coli* isolated from chicken meat showing resistance against TE, 75.6% against CXM, and 42.8% against SXT [37]. Our results indicate a similarly high frequency of AMR against AMP, TE, and SXT resistance, which is comparable to the findings of Güven and Kizil [4] and Önen et al. [37]. However, Inat et al. [38] reported lower frequencies of AMR in *E. coli* isolates originating from Samsun in Türkiye. Merely 15% of the *E. coli* strains were resistant to AMP, 11.3% to CN, 10% to C, and 8.8% to IMP. Interestingly, meropenem and/or imipenem resistance was detected in *E. coli* strains in recent studies [4,38], which has not been reported before [3,37].

Among the 18 *E. coli* strains in our study that tested positive for ESBL by DDST, 17 (94.4%) exhibited MDR (Table 7). In detail, 17 strains contained *bla*_TEM_, 12 strains contained *bla*_CTX-M_, 1 strain contained *bla*_SHV_, and 1 strain had *bla*_OXA_. Other workers found only 4 of 18 ESBL-producing isolates using the DDST test [38]. This difference can be explained by the fact that *bla*_TEM_ was detected in 97% of the *E. coli* strains in our study. In our study, 78% of the *E. coli* strains were AMP-resistant since they contained the *bla*_TEM_ gene, which is one of the main genes responsible for AMP resistance. The high frequency of the *bla*_TEM_ gene, which is one of the ESBL genes, is contrasted with the low percentage of phenotypic ESBL (17.8%). Similarly, phenotypic ESBL production was less frequent than the presence of *bla*_CTX-M_ (45.5%), which is the most important ESBL gene, indicating that the relationship between genotypic and phenotypic beta-lactamase production needs to be investigated. Similar to our study, Inat et al. [38] reported that the presence of genotypic ESBL genes was more frequent than phenotypic ESBL production. It is important to note that both studies investigated phenotypic ESBL production by using the double disc synergy test.

### 3.3. Genotypic Determination of Antibiotic Resistance Genes in E. coli Strains

The increase in combined resistance to multiple antimicrobial groups and the high proportion of ESBL-producing *E. coli* is of concern, as this limits treatment options for patients suffering from infections caused by these bacteria [39]. ESBL-producing *E. coli* strains and their ESBL types have been studied in poultry production worldwide [12,13,14,40]. However, there are relatively limited data in Türkiye on ESBL-producing *E. coli* from chicken meat [3,4,38,41]. There is a need to understand better the epidemiology of ESBL-producing bacteria in chicken meat. With respect to consumers’ exposure, big cities and tourist destinations can be critical. Istanbul is a metropolis and the biggest city in terms of population and economy in Türkiye. Istanbul hosted almost 17.5 million tourists and became the most visited tourist destination place in the world in 2023 [42]. In this context, Istanbul is an import and export center and transport corridor between Europe, Asia, and the Middle East.

We detected the *bla*_TEM_ gene in 98 out of 101 strains (97.02%), the *bla*_CTX-M_ gene in 46 strains (45.5%), the *bla*_SHV_ gene in 10 strains (9.9%), and the *bla*_OXA_ gene in only 1 strain (0.9%). None of the 101 *E. coli* strains contained the *mcr*-1, *mcr*-2, *mcr*-3, *mcr*-4, *mcr*-5, *mcr*-6, *mcr*-7, and *mcr*-8, *bla*_NDM_, *bla*_KPC_, *bla*_VIM_, or *bla*_OXA-48_ genes.

In Germany, 185 ESBL-producing *E. coli* were found in 175 of 399 chicken meat samples [43]. ESBL genes were identified by multiplex PCR: *bla*_TEM-52_ (*n* = 16), *bla*_CTX-M-1_ (*n* = 77), and *bla*_SHV-12_ (*n* = 82). The percentage of *bla*_CTX-M-1_ was similar to that in our study. Such a high incidence of *bla*_CTX-M-1_ in Berlin, the most populated city in Germany, and Istanbul, the largest city in Türkiye, indicate that this gene threatens large populations in different geographical areas.

In Türkiye, 152 *E. coli* strains were analyzed by combining disk diffusion tests, and 28 (18.4%) strains were found to produce ESBL [41]. In another study in Türkiye, 100 chicken meat samples were collected, and 214 *E. coli* isolates were cultivated from 72 samples positive for *E. coli* [44]. Five strains (2.3%) were phenotypically ESBL, whereas PCR analyses detected *bla*_TEM_, *bla*_SHV_, and *bla*_CTX-M_ in 8, 3, and 7 *E. coli* strains, respectively. In the present study, the frequency of ESBL-producing *E. coli* from chicken meat was significantly higher than that reported by Çil et al. [41] and Bilge et al. [44]. In particular, there was a high increase in the frequency of *bla*_TEM_ but no significant difference in the rate of phenotypic ESBL-producing *E. coli*. Many ESBL-related genes have been discovered in recent years. This study contributes to a better understanding of the ESBL-producing *E. coli* epidemiology in Türkiye and provides important data for future studies.

Our study showed no evidence of *E. coli* strains carrying *mcr* and carbapenem resistance genes. However, similar studies are reporting on these extremely important antimicrobials. Randall et al. [45] did not detect colistin- and carbapenem-resistant *E. coli* isolates in 622 chicken meat samples; all ESBL-producing *E. coli* isolates were sensitive to meropenem and colistin. The striking difference to our study is that *bla*_CTX-M-1_ was the dominant gene in the isolates tested by Randall et al. [45], whereas the *bla*_TEM-1_ gene was commonly observed in our study. We detected meropenem-resistant *E. coli* isolates, especially in samples from the European side of Istanbul, whereas *bla*_NDM_, *bla*_KPC_, *bla*_VIM_, and *bla*_OXA-48_ genes related to this resistance were not detected. It should be noted that carbapenem resistance genes were not detected by PCR either in our study or in that of Randall et al. (2021). However, Randall et al. [45] used CHROMagar KPC in their carbapenem resistance study, which may account for some differences.

### 3.4. Distribution of MDR in E. coli Strains

The number of MDR bacteria is increasing, and the foodborne transfer of antimicrobial resistance is an important issue. Bacteria harboring resistance genes may originate from (food-producing) animals or be present in foods due to cross-contamination, thus threatening public health. Increased numbers of infections with bacteria and antibiotic-resistant genes complicate treatment [46,47].

Baran et al. [3] detected MDR in 99 (94.29%) *E. coli* strains in Türkiye. All *E. coli* isolates were sensitive to meropenem. Conversely, the rate of MDR (79.2%) was lower than our study. However, the higher rate of meropenem resistance (34%) is of concern. This difference may have been caused by the fact that our study was conducted in Istanbul, where chicken meat from many production points in Türkiye is available, whereas Baran et al. [3] tested samples in Erzurum province, which mostly reflects the production in eastern Anatolia.

Many *E. coli* strains (80/101; 79.1%) demonstrated MDR in this study. Antibiotic resistance against AMC (88/101; 87.1%) was higher than against the other antimicrobials studied. No isolate showed resistance against TZP. In a study conducted in Brazil, 200 swabs were taken from broilers, and 13 ESBL-producing *E. coli* strains were found as an MDR [48]. In addition, Ferreira et al. [48] identified 16 ESBL-producing *Enterobacteria* isolates. Among them, 13 isolates were *E. coli*. The frequency of resistance to CN, C, and SXT was similar to that reported in our study, whereas resistance against CIP was much more frequent (15/16, 93.7%). Considering the contribution of chicken meat to human diets and exposure to raw chicken meat during food processing, MDR in *E. coli* isolated from chicken is a serious health concern.

### 3.5. Biofilm Formation of E. coli and ESBL-Producing E. coli Strains

The results of the biofilm assay indicate that *E. coli* strains can form more biofilm in TSB media containing 1% (*w*/*v*) sucrose (*n* = 44) than in other media. Moreover, the highest number of biofilm-producing *E. coli* strains was isolated in the wing part of a chicken meat sample from the European side with a value of 3223 at 595 nm in TSB containing a 0.6% (*w*/*v*) yeast extract (Table 8). There are 7 *E. coli* strains in TSB as follows: 6 *E. coli* strains in TSB containing 1% (*w*/*v*) sucrose, 5 *E. coli* strains in TSB containing 0.6% (*w*/*v*) yeast extract, 3 E. coli strains in BHI, and 1 *E. coli* strain in NB-produced biofilms. Genotypic ESBL-producing *E. coli* strains demonstrated their ability to produce biofilms with 38 strains in TSB, 50 strains in TSB containing 1% (*w*/*v*) sucrose, 34 strains in TSB containing 0.6 % (*w*/*v*) yeast extract, 18 strains in BHI, 5 strain in NB and 4 strains in LB.

The formation of biofilms by bacterial strains is a concern in both food crops and food processing facilities. The production of biofilms by MDR bacteria is a major concern in the food chain. Limited data on biofilm-producing *E. coli* strains in chicken meat samples are available worldwide. In Brazil, 150 samples were collected from the largest chicken meat exporter, and 88 *E. coli* strains were found [40]. Among these strains, 84 (56%) could produce biofilms, as assessed by the microplate method. In addition, 17.04% of the *E. coli* strains were capable of ESBL production. The authors [40] used only TSB as a medium to study biofilm formation, whereas six different media were used in our study. The present study obtained results similar to those of Crecencio et al. [40], with *bla*_TEM-1_ (73.3%) being the most common gene found in their analysis of ESBL-producing *E. coli*. The significant difference between the two studies was the rate of *bla*_SHV-1_. They reported the presence of *bla*_SHV-1_ in 46.6% of the bacterial isolates in 2020, while the frequency observed in this study was 9.9%. Similar results in samples from two different continents demonstrate the importance of biofilm-forming *E. coli* isolates. These isolates, which are more resistant to environmental conditions, threaten global health. The high level of plasmid-origin gene transfection in both studies indicates that ESBL-producing bacteria will continue to increase.

## 4. Conclusions

In the food chain, there are several concerns about MDR in bacteria. Poultry meat products carry different antibiotic resistance genes, including those conferring resistance against critical last-resort antibiotics, such as colistin. The present study found MDR, biofilm-producing, and ESBL-producing *E. coli* strains in chicken meat that retailed in Istanbul, Türkiye. Among these isolates, *bla*_TEM-1_ was the dominant ESBL gene. We conclude that chicken meat is an important reservoir for ESBL-producing *E. coli*. The production of biofilms by these bacteria is a challenge for maintaining hygiene throughout the food chain. Biofilm-producing *E. coli* isolates can easily transfer antibiotic-resistant genes and survive difficult conditions. Fortunately, our results indicate the absence of mobilized colistin and carbapenem resistance genes. More studies are needed on ESBL-producing *E. coli*. In order to fill this knowledge gap, this study provides data on ESBL-producing *E. coli* strains originating from chicken meat in the European and Asian parts of the Istanbul metropolis and the determination to produce biofilm-formation-isolated ESBL-producing *E. coli* strains.

## Figures and Tables

**Table 2:** Primers for the detection of different carbapenem resistance genes by PCR.

Target Gene	Primer Sequence (5′→3′)	Melting Temperature T_m_ (°C)	Product Size (bp)
*bla*_OXA-48_	OXA_F 5′-TTGGTGGCATCGATTATCGG-3′ OXA_R 5′-GAGCACTTCTTTTGTGATGGC-3′	58	744
*bla*_NDM_	NDM_F 5′-TGGCAGCACACTTCCTATC-3′ NDM_R 5′-AGATTGCCGAGCGACTTG-3′	58	488
*bla*_KPC_	KPC_F 5′-CTGTCTTGTCTCTCATGGCC-3′ KPC_R 5′-CCTCGCTGTRCTTGTCATCC-3′	60	796
*bla*_VIM_	VIM_F: 5′-AGTGGTGAGTATCCGACAG-3′ VIM_R: 5′-TCAATCTCCGCGAGAAG-3′	52	212

**Table 3:** Primers were used to detect different mobilized colistin resistance genes (1–5) by multiplex PCR.

Target Gene	Primer Sequence (5′→3′)	Melting Temperature T_m_ (°C)	Product Size (bp)
*mcr*-1	AGTCCGTTTGTTCTTGTGGC AGATCCTTGGTCTCGGCTTG	58	320
*mcr*-2	CAAGTGTGTTGGTCGCAGTT TCTAGCCCGACAAGCATACC	58	715
*mcr*-3	AAATAAAAATTGTTCCGCTTATG AATGGAGATCCCCGTTTTT	58	929
*mcr*-4	TCACTTTCATCACTGCGTTG TTGGTCCATGACTACCAATG	58	1116
*mcr*-5	ATGCGGTTGTCTGCATTTATC TCATTGTGGTTGTCCTTTTCTG	58	1644

**Table 4:** Primers used for the detection of different mobilized colistin resistance genes by monoplex PCR.

Target Gene	Primer Sequence	Melting Temperature T_m_ (°C)	Product Size (bp)
*mcr*-6	MCR-6F 5′-GTCCGGTCAATCCCTATCTGT-3′ MCR-6R 5′-ATCACGGGATTGACATAGCTAC-3′	55	556
*mcr*-7	MCR-7F 5′-TGCTCAAGCCCTTCTTTTCGT-3′ MCR-7R 5′-TTCATCTGCGCCACCTCGT-3′	55	892
*mcr*-8	MCR-8F 5′-AACCGCCAGAGCACAGAATT-3′ MCR-8R 5′-TTCCCCCAGCGATTCTCCAT-3′	60	667

**Table 5:** Antibiotic susceptibility of *E. coli* strains.

Antibiotic Group	Name of Antibiotic	Distribution of *E. coli* Isolates According to CLSI [23]		Distribution of *E. coli* Isolates According to EUCAST [22]	
		R (%)	S (%)	R (%)	S (%)
Aminoglycoside	Amikacin 30 µg	0% (*n* = 0)	100% (*n* = 101)	6.9% (*n* = 7)	93.7% (*n* = 94)
	Gentamicin 10 µg	20.7% (*n* = 21)	79.3% (*n* = 80)	14.8% (*n* = 15)	85.2% (*n* = 86)
Cephalosporins	Cefotaxime 30 µg	35.6% (*n* = 36)	74.4% (*n* = 65)	17.8% (*n* = 18)	82.2% (*n* = 83)
	Cefoxitin 30 µg	5.9% (*n* = 6)	94.1% (*n* = 95)	11.8% (*n* = 11)	88.2% (*n* = 90)
	Ceftazidime 30 µg	10.8% (*n* = 11)	89.2% (*n* = 90)	10.8% (*n* = 11)	89.2% (*n* = 90)
	Cefuroxime 30 µg	19.8% (*n* = 20)	80.2% (*n* = 81)	26.7% (*n* = 27)	73.3% (*n* = 74)
Carbapenems	Meropenem 10 µg	34.6% (*n* = 35)	65.4% (*n* = 66)	33.6% (*n* = 34)	66.4% (*n* = 67)
Fluoroquinolones	Ciprofloxacin 5 µg	45.5% (*n* = 46)	54.5% (*n* = 55)	45.5% (*n* = 46)	54.5% (*n* = 55)
Monobactam	Aztreonam 30 µg	15.8% (*n* = 16)	84.2% (*n* = 85)	17.8% (*n* = 18)	82.2% (*n* = 83)
Nitrofuran	Nitrofurantoin 300 µg	1.9% (*n* = 2)	98.1% (*n* = 99)	0.9% (*n* = 1)	99.1% (*n* = 100)
Penicillin	Ampicillin 10 µg	78.2% (*n* = 79)	21.8% (*n* = 22)	78.2% (*n* = 79)	21.8% (*n* = 22)
	Amoxicillin clavulanic acid 30 µg	67.3% (*n* = 68)	32.7% (*n* = 33)	87.1% (*n* = 88)	12.9% (*n* = 13)
	Piperacillin-tazobactam 30 µg	0% (*n* = 0)	100% (*n* = 101)	0% (*n* = 0)	100% (*n* = 101)
Phenicol	Chloramphenicol 30 µg	59.4% (*n* = 60)	41.5% (*n* = 51)	59.4% (*n* = 60)	41.5% (*n* = 51)
Sulfonamides	Trimethoprim-Sulfamethoxazole 25 µg	52.4% (*n* = 53)	47.5% (*n* = 48)	52.4% (*n* = 53)	47.5% (*n* = 48)
Tetracyclines	Tetracycline 30 µg	74.2% (*n* = 75)	25.8% (*n* = 26)	*	*

* EUCAST [22] does not provide a breakpoint value for this antibiotic.

**Table 6:** Distribution of antibiotic-resistant *E. coli* strains according to place of sampling (European or Asian part) in Istanbul.

Antibiotic Group	Name of Antibiotic	Distribution of *E. coli* Isolates According to CLSI [23]		Distribution of *E. coli* Isolates According to EUCAST [22]	
		European Side R (%) (*n* = 58)	Asian Side R (%) (*n* = 43)	European Side R (%) (*n* = 58)	Asian Side R (%) (*n* = 43)
Aminoglycoside	Amikacin 30 µg	0% (*n* = 0)	0% (*n* = 0)	3.4% (*n* = 2)	11.6% (*n* = 5)
	Gentamicin 10 µg	18.9% (*n* = 11)	23.2% (*n* = 10)	12.1% (*n* = 7)	18.6% (*n* = 8)
Cephalosporins	Cefotaxime 30 µg	41.3% (*n* = 24)	27.9% (*n* = 12)	18.8% (*n* = 10)	18.6% (*n* = 8)
	Cefoxitin 30 µg	1.7% (*n* = 1)	11.6% (*n* = 5)	8.6% (*n* = 5)	16.2% (*n* = 7)
	Ceftazidime 30 µg	5.1% (*n* = 3)	18.6% (*n* = 8)	5.1% (*n* = 3)	18.6% (*n* = 8)
	Cefuroxime 30 µg	18.9% (*n* = 11)	20.9% (*n* = 9)	20.6% (*n* = 12)	34.8% (*n* = 15)
Carbapenems	Meropenem 10 µg	53.4% (*n* = 31)	9.3% (*n* = 4)	51.7% (*n* = 30)	9.3% (*n* = 4)
Fluoroquinolones	Ciprofloxacin 5 µg	48.2% (*n* = 28)	41.8% (*n* = 18)	48.2% (*n* = 28)	41.8% (*n* = 18)
Monobactam	Aztreonam 30 µg	13.7% (*n* = 8)	18.6% (*n* = 8)	13.7% (*n* = 8)	18.6% (*n* = 10)
Nitrofuran	Nitrofurantoin 300 µg	0% (*n* = 0)	4.6% (*n* = 2)	0% (*n* = 0)	2.3% (*n* = 1)
Penicillin	Ampicillin 10 µg	82.7% (*n* = 48)	72.09% (*n* = 31)	82.7% (*n* = 48)	72.09% (*n* = 31)
	Amoxicillin clavulanic acid 30 µg	62.06% (*n* = 36)	74.4% (*n* = 32)	79.3% (*n* = 46)	97.6% (*n* = 42)
	Piperacillin-tazobactam 30 µg	0% (*n* = 0)	0% (*n* = 0)	0% (*n* = 0)	0% (*n* = 0)
Phenicol	Chloramphenicol 30 µg	51.7% (*n* = 30)	69.7% (*n* = 30)	51.7% (*n* = 30)	69.7% (*n* = 30)
Sulfonamid	Trimethoprim-Sulfamethoxazole 25 µg	53.4% (*n* = 31)	51.1% (*n* = 22)	53.4% (*n* = 31)	51.1% (*n* = 22)
Tetracyclines	Tetracycline 30 µg	74.1% (*n* = 43)	74.4% (*n* = 32)	*	*

* EUCAST [22] does not provide a breakpoint value for this antibiotic.

**Table 7:** Antibiotic resistance of *E. coli* isolates phenotypically producing ESBL using the DDTS test.

*E. coli* Isolates Phenotypically Producing ESBL	CLSI [23]			EUCAST [22]	
	On Which Side of Istanbul the Sample Was Collected	*E. coli* Isolates Resistant to Two Antibiotics	Resistant to How Many Groups of Antibiotics?	*E. coli* Isolates Resistant to One Antibiotic	Resistant to How Many Groups of Antibiotics?
S007	Europe	-	6 (AMC,ATM,CTX,CAZ, SXT,TE,C,CXM,AMP)	-	5 (AMC,ATM,CTX,CAZ,SXT, C,CXM,AMP)
S024	Europe	-	3 (CTX,CIP,CXM,AMP)	-	3 (CTX,CIP,CXM,AMP)
S064	Europe	-	5 (CTX,CIP,TE,C,CXM, AMP)	-	4 (CTX,CIP,TE,C,CXM,AMP)
S074	Europe	2 (SXT, TE)	-	1 (SXT)	-
S081	Europe	-	6 (AMC,ATM,CTX,CIP,TE, C,CXM,AMP)	-	5 (AMC,ATM CTX,CIP,TE,C, CXM,AMP)
S086	Europe	-	7 (AMC,ATM,SXT,CTX, CIP, CN, C, CXM, AMP)	-	7 (AMC,ATM,CTX,CIP,CN,C, CXM, AMP)
S090	Europe	-	6 (AMP,ATM,CTX,SXT,TE,C,CXM,AMP)	-	5 (AMP,ATM,CTX,CAZ,SXT,C,CXM,AMP)
S092	Europe	-	6 (AMP,ATM,CTX,SXT,FOX,TE,C,CXM,AMP)	-	5 (AMP,ATM,CTX,SXT,FOX,C,CXM,AMP)
S100	Europe	-	7 (AMC,CTX,SXT,CN,CIP,TE,C,AMP)	-	6 (AMC,CTX,SXT,CN,CIP,FOX,C,CXM,AMP)
S115	Asia	-	8 (AMC,ATM,CTX,CAZ,SXT,CN,TE,C,CXM,AMP,MEM)	-	7 (AMC,ATM,CTX,CAZ,SXT,CN,C,CXM,AMP,MEM)
S116	Asia	-	7 (AMC,ATM,CTX,SXT,CN,TE,C,CXM,AMP)	-	6 (AMC, ATM,CTX,SXT,CN,C,CXM,AMP)
S118	Asia	-	9 (AMC,ATM,CTX,CAZ,CIP,SXT,CN,TE,C,CXM,AMP,MEM)	-	8 (AMC,ATM,CTX,CAZ,CIP,SXT,CN,C,AK,CXM,AMP,MEM)
S127	Asia	-	8 (AMC,ATM,CTX,SXT,CN,TE,C,CXM,AMP,MEM)	-	7 (AMC, ATM,CTX,SXT,CN,C,CXM,AMP,MEM)
S128	Asia	-	8 (AMC,ATM,CTX,SXT,CIP,TE,C,CXM,AMP,MEM)	-	7 (AMC, ATM,CTX,SXT,CIP,C,CXM,AMP,MEM)
S136	Asia	-	6 (AMC,ATM,CTX,TE,C,CXM,AMP,MEM)	-	5 (AMC,ATM,CTX,C,CXM,AMP,MEM)
S152	Asia	-	6 (AMC,CAZ,FOX,TE,C,CXM,AMP,MEM)	-	5 (AMC,CAZ,FOX,C,CXM,AMP,MEM)
S174	Asia	-	8 (AMC,ATM,CTX,SXT,CN,TE,C,CXM,AMP,MEM)	-	7 (AMC,ATM,CTX,CAZ,SXT,CN,C,CXM,AMP,MEM)
S191	Asia	-	9 (AMC,ATM,CAZ,CTX,CIP,SXT,CN,TE,C,CXM,AMP,MEM)	-	8 (AMC,ATM,CAZ,CTX,CIP,SXT,CN,C,CXM,AMP,MEM)

**Table 8:** Biofilm-producing *E. coli* number and distribution.

Medium	Number of Biofilm-Producing *E. coli* Isolated from Samples Collected in the European Side of Istanbul	Number of Biofilm-Producing *E. coli* Isolated from Samples Collected in the Asian Side of Istanbul
TSB	17 (29.3%)	12 (27.9%)
BHI	8 (13.7%)	3 (6.9%)
NB	3 (5.1%)	2 (4.6%)
LB	3 (5.1%)	1 (2.3%)
1% sucrose TSB	32 (55.1%)	12 (27.9%)
0.6% yeast extract TSB	19 (32.7%)	12 (27.9%)

## Data Availability

The data presented in this study are available on request from the corresponding authors.
